# Hybrid zone 1 repair of a giant post-traumatic intrathoracic left subclavian artery pseudoaneurysm

**DOI:** 10.1016/j.jvscit.2026.102319

**Published:** 2026-05-14

**Authors:** Erasmia El Kanty, Natasha Hasemaki, Antonia Skotsimara, Michail Tsotsios, Ilias Avgerinos, Athanasios Katsargyris, Chris Klonaris

**Affiliations:** a 2nd Vascular Surgery Department, National and Kapodistrian University of Athens, Laikon General Hospital of Athens, Athens, Greece; b Department of Vascular Surgery, University Hospital LMU Munich, Munich, Germany

**Keywords:** Subclavian artery pseudoaneurysm, Post-traumatic pseudoaneurysm, Hybrid repair, Thoracic endovascular aortic repair (TEVAR), Supra-aortic debranching, Clavicular fracture

## Abstract

Subclavian artery pseudoaneurysms are rare and may present decades after blunt trauma. We report the case of a 38-year-old man presenting 22 years after clavicular fracture with a giant intrathoracic pseudoaneurysm of the proximal left subclavian artery, with the vertebral artery arising immediately distal to the lesion. Due to the limited proximal landing zone and short carotid-subclavian distance, a one-stage hybrid repair was performed, consisting of carotid-carotid-subclavian bypass and zone 1 thoracic endovascular aortic repair. The procedure achieved complete exclusion with preserved cerebral and upper limb perfusion. At 5 years, the patient remains asymptomatic without endoleak or graft-related complications.

Subclavian artery aneurysms (SAAs) are relatively rare, accounting for approximately 1% to 2% of all peripheral arterial aneurysms.^1^^,^^2^ True SAAs most often arise from atherosclerosis or thoracic outlet syndrome, whereas subclavian artery pseudoaneurysms (SAPs) are typically iatrogenic, traumatic, or infection related. Both SAAs and SAPs may be further classified as intrathoracic or extrathoracic.^3^^,^^4^ Surgical or endovascular treatment of subclavian artery pathologies is inherently challenging due to the proximity to the aortic arch and supra-aortic branches, and the close relationship with critical branches such as the vertebral artery and the internal mammary artery. These anatomical constraints necessitate careful, individualized treatment planning.

Until the early 1990s, open surgical repair was the standard therapeutic approach, but was associated with significant morbidity, including nerve injury and perioperative bleeding.^4^^,^^5^ Adequate exposure frequently requires sternotomy or thoracotomy, increasing perioperative risk due to extensive surgical dissection and proximity to critical neurovascular structures.

In the contemporary era, many SAAs can be treated using minimally invasive endovascular or hybrid techniques, offering technical success with reduced perioperative morbidity. Endovascular management has increasingly been adopted for SAAs and SAPs, providing lower complication rates and a less invasive alternative to open surgery.^1^

The choice of treatment strategy for SAAs is largely dictated by anatomical factors, particularly lesion location and the availability of adequate proximal and distal landing zones. Lesions arising distal to the origin of the vertebral artery with sufficient landing zones may be amenable to isolated endovascular repair with stent graft deployment. In contrast, aneurysms involving the proximal subclavian artery or located in close proximity to the origin of the vertebral artery may lack adequate sealing zones, precluding isolated subclavian stent grafting. In such cases, extension of the repair into the aortic arch and descending aorta, combined with supra-aortic revascularization, may be required to achieve durable aneurysm exclusion while preserving cerebral and upper limb perfusion. In this context, we present a case illustrating the role of hybrid repair in the management of a giant intrathoracic post-traumatic left SAP. Written informed consent was obtained from the patient for publication of this case report and the accompanying images.

## Case report

**Fig 1:**
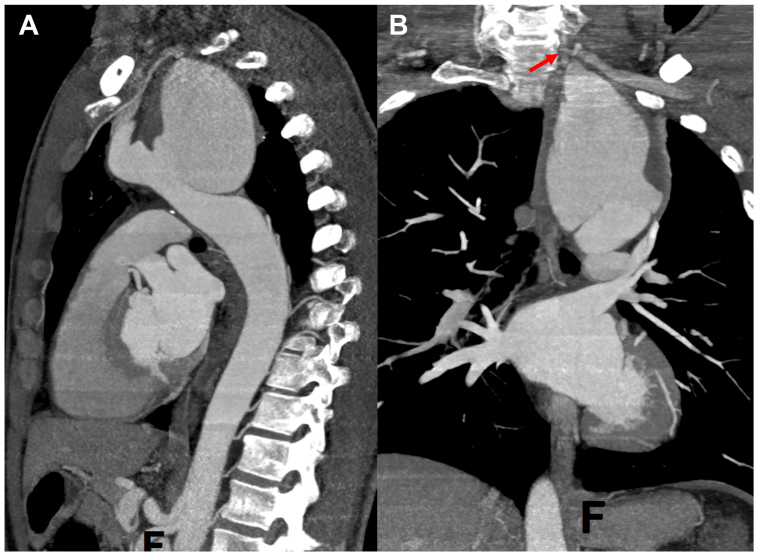
**(A)** Sagittal reconstruction of computed tomography angiography (CTA) demonstrating a giant intrathoracic pseudoaneurysm arising from the proximal left subclavian artery (LSA). **(B)** Coronal reconstruction showing the close proximity of the pseudoaneurysm to the origin of the left vertebral artery (*red arrow*).

**Fig 2:**
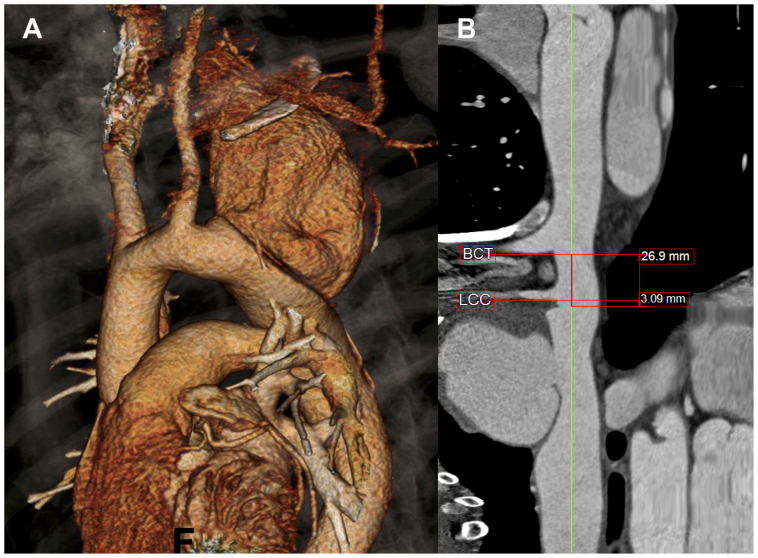
**(A)** Three-dimensional reconstruction demonstrating the giant proximal left subclavian artery (LSA) pseudoaneurysm (SAP) in relation to the aortic arch branches. **(B)** Centerline reconstruction illustrating the short distance between the LSA and left common carotid artery (*LCC*), precluding an adequate proximal landing in zone 2. *BCT*, Brachiocephalic trunk.

A 38-year-old man presented to our department with a progressively enlarging pulsatile mass in the left supraclavicular and infraclavicular region, associated with local pain and a sensation of pressure in the supraclavicular area. His medical history was notable for a left clavicular fracture sustained 22 years earlier, which was managed conservatively. There was no history of recent trauma, prior vascular intervention, fever, or systemic symptoms. On physical examination, a prominent pulsatile mass was palpable in the left supraclavicular region. Distal pulses were present, and there were no signs of upper extremity ischemia or neurological deficit. Computed tomography angiography (CTA) revealed a giant pseudoaneurysm arising from the proximal left subclavian artery (LSA), measuring as large as 103 mm in maximum diameter (Fig 1, *A*); the left vertebral artery originated immediately distal to the aneurysm (Fig 1, *B*). Review of the CTA revealed no evidence of bony callus formation or thoracic outlet compression contributing to the lesion. Preoperative CTA demonstrated a complete circle of Willis, and both vertebral arteries were of similar caliber, with no evidence of dominance. Laboratory investigations, including inflammatory markers, were within normal limits, and blood cultures were negative. Given the absence of clinical or laboratory evidence of infection and the history of prior trauma, the pseudoaneurysm was considered to be of delayed post-traumatic origin. Given the large size of the aneurysm, its location, the need to preserve cerebral and upper limb perfusion, and the symptomatic presentation, a one-stage hybrid approach was planned, combining supra-aortic debranching and thoracic endovascular aortic repair. A total endovascular solution was not feasible, because no off-the-shelf branched device was available at the time and the acute onset precluded waiting for a custom-made device. Due to the short distance between the origins of the left common carotid artery (CCA) and the subclavian artery, an adequate proximal sealing zone in zone 2 could not be achieved; therefore, a proximal landing in zone 1 was required (Fig 2).

**Fig 3:**
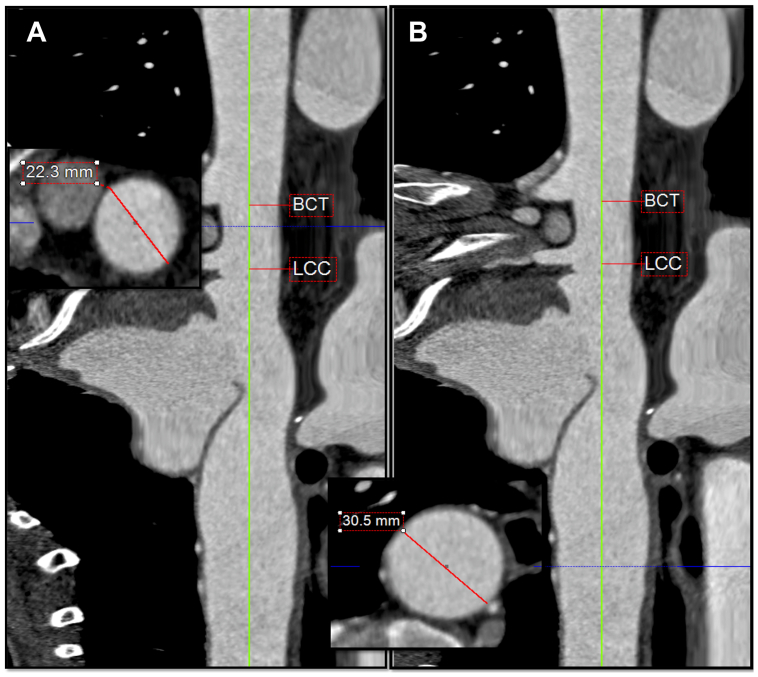
Multiplanar computed tomography angiography (CTA) reconstructions demonstrating the discrepancy between the proximal **(A)** and distal **(B)** aortic diameters at the intended landing zones. The proximal landing zone measured 22 mm, whereas the distal thoracic aorta measured approximately 30 mm. This diameter mismatch necessitated the implantation of two overlapping thoracic stent grafts of different diameters to ensure optimal apposition and adequate proximal and distal sealing. *BCT*, Brachiocephalic trunk; *LCC*, left common carotid.

Under general anesthesia, cervical exposures were performed through a right cervical incision for access to the right CCA and a left supraclavicular incision, allowing exposure of both the left CCA and the LSA. After systemic heparinization, supra-aortic debranching was performed using a 7-mm Dacron graft, based on size matching with the CCAs, which measured approximately 7 mm in diameter. An end-to-side anastomosis was created to the right CCA, the left CCA was subsequently reimplanted onto the graft in an end-to-side fashion with ligation of its proximal segment, and the graft was then extended to the LSA with an end-to-side anastomosis. The proximal LSA was ligated proximal to the origin of the vertebral artery. After surgical debranching, bilateral common femoral arterial access was obtained. Thoracic endovascular repair was performed using two overlapping Gore TAG thoracic stent grafts (W. L. Gore & Associates), measuring 26 × 26 × 100 mm proximally and 34 × 34 × 150 mm distally, selected to accommodate the different proximal and distal aortic diameters (Fig 3). Completion CTA demonstrated complete exclusion of the pseudoaneurysm, patency of the bypass graft, and no evidence of endoleak. The postoperative course was uneventful, with no neurological deficits, upper limb ischemia, or access-related complications. The patient was discharged on postoperative day 5 on dual antiplatelet therapy, given his young age and low bleeding risk, to optimize long-term bypass patency. Dual antiplatelet therapy was continued for 6 months, followed by long-term single antiplatelet therapy with clopidogrel.

**Fig 4:**
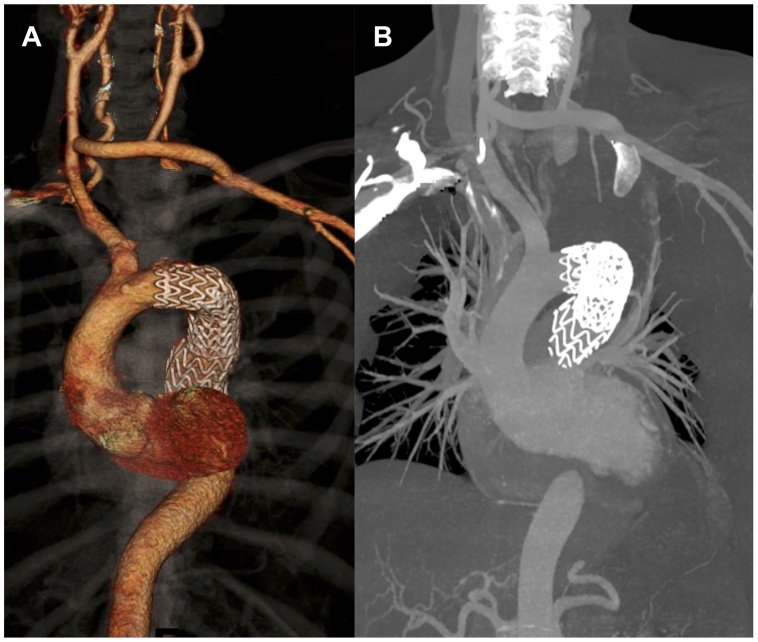
**(A)** Three-dimensional computed tomography angiography (CTA) reconstruction at the 5-year follow-up demonstrating durable exclusion of the pseudoaneurysm and configuration of the thoracic stent graft. **(B)** Coronal reconstruction showing patency of the carotid-carotid-subclavian bypass, preserved supra-aortic flow, and absence of endoleak or device-related complications.

At the 1-month follow-up, CTA confirmed optimal stent graft apposition, complete exclusion of the pseudoaneurysm, preserved flow through the supra-aortic branches, and absence of endoleak. At the 5-year follow-up, the patient remained in good general condition and asymptomatic, with preserved left upper limb perfusion, durable patency of the carotid-carotid-subclavian bypass on duplex ultrasound examination, and no evidence of endoleak, stent migration, or graft-related complications on CTA (Fig 4).

## Discussion

The present report describes a giant intrathoracic post-traumatic left SAP presenting more than two decades after clavicular fracture, successfully managed with a one-stage hybrid zone 1 repair and demonstrating excellent 5-year durability. Ferreira et al^6^ highlight that late clinical presentation, often decades after the inciting event, is characteristic of these lesions and typically manifests with pain, swelling, or compressive symptoms rather than acute hemorrhage. The present case mirrors this pattern, with pseudoaneurysm formation occurring more than two decades after a clavicular fracture.

The pathophysiology of delayed post-traumatic pseudoaneurysm formation is believed to involve chronic arterial wall degeneration caused by repetitive mechanical stress, local fibrosis, or bony callus formation at the fracture site. Similar delayed presentations after clavicular fractures have been reported in the literature, underscoring the need for long-term clinical vigilance in patients with a history of trauma to the thoracic outlet region.^4^^,^^6^

Management of proximal SAPs remains technically demanding because of their deep anatomical location and proximity to the aortic arch and critical branches such as the vertebral artery and internal mammary artery. Although these branches are essential, their sacrifice may be considered in selected cases depending on collateral circulation and individual anatomical factors; however, preservation is generally preferred whenever feasible. Traditional open surgical repair often requires extensive exposure through sternotomy, clavicular resection, or combined cervicothoracic approaches, which are associated with considerable morbidity, including nerve injury, hemorrhage, and prolonged recovery.^7^ Ferreira et al^6^ emphasize that open repair is particularly challenging when pseudoaneurysms arise from the proximal one-third of the vessel, frequently necessitating major surgical exposure to achieve proximal control.

Endovascular techniques have increasingly been adopted as a less invasive alternative, demonstrating favorable short-term and midterm outcomes with reduced perioperative morbidity. Several series and case reports have shown that covered stent placement can successfully exclude SAPs when adequate landing zones are available and vessel tortuosity is limited.^1^ However, purely endovascular repair may be contraindicated in cases with insufficient proximal landing zones, involvement of major arterial branches, or complex anatomy.

Hybrid approaches combining supra-aortic debranching with thoracic endovascular aortic repair have emerged as a valuable solution in such complex scenarios. By revascularizing critical branches before stent graft deployment, hybrid repair enables secure proximal sealing while preserving cerebral and upper limb perfusion. This strategy is particularly advantageous in patients with short distances between the left CCA and LSA, where a zone 2 landing is not feasible, as observed in the present case. Similar hybrid strategies have been described with favorable outcomes in anatomically challenging SAPs.^8^ Reported outcomes of endovascular and hybrid repair for SAAs and pseudoaneurysms are generally favorable. Available evidence, largely derived from small case series and case reports, suggests high technical success rates with low perioperative morbidity and satisfactory midterm durability.^1^^,^^3^^,^^4^^,^^8^^,^^9^ However, robust long-term data remain limited due to the rarity of this condition.

In recent years, advanced total endovascular strategies using fenestrated or branched thoracic endovascular aortic repair have been introduced to enable cervical vessel preservation while avoiding open supra-aortic debranching and its associated morbidity. These techniques offer a promising minimally invasive alternative, particularly in complex arch pathology. However, their application in SAAs and pseudoaneurysms remains limited, primarily due to the rarity of this condition and the resulting lack of robust evidence and standardized device configurations. Additionally, at the time of treatment, no off-the-shelf branched thoracic endografts were commercially available in Europe.

Although total endovascular arch repair with branched or fenestrated devices has expanded significantly in recent years, increasing the feasibility of entirely endovascular supra-aortic vessel preservation, these technologies remain limited by availability, manufacturing time, anatomical constraints, and the need for advanced technical expertise. Moreover, in acute or urgent clinical scenarios, the time required for custom device production may render such strategies impractical. In this context, cervical debranching combined with thoracic endovascular aortic repair continues to represent a reliable and reproducible solution, offering immediate applicability, durable proximal sealing, and controlled supra-aortic revascularization, particularly in anatomically complex cases.

In the present case, a total endovascular approach was considered but deemed unsuitable primarily due to the unavailability of an off-the-shelf branched device and the acute clinical presentation, which precluded waiting for the manufacture of a custom-made graft. Additionally, the absence of an adequate distal landing zone within the LSA further limited the feasibility of a fully endovascular strategy. The close proximity of the pseudoaneurysm to the origin of the vertebral artery precluded safe deployment of a bridging stent. Even with vertebral artery revascularization, the required bridging stent length would have been extensive, potentially increasing the risk of target vessel instability, kinking, and long-term durability concerns. For these reasons, a hybrid approach was favored as the most anatomically sound and durable solution.

The present case supports the growing body of evidence advocating individualized treatment planning based on anatomical characteristics, clinical presentation, and patient-specific factors. As highlighted by Ferreira et al,^6^ no single therapeutic approach is universally applicable, and the selection of open, endovascular, or hybrid repair should be tailored accordingly. In this context, the present case further underscores the continued relevance of cervical debranching in the contemporary endovascular era, particularly in urgent scenarios where total endovascular solutions may not be feasible. In our patient, a one-stage hybrid repair allowed complete exclusion of a giant intrathoracic pseudoaneurysm with preservation of supra-aortic and upper limb perfusion and excellent midterm durability. Although the long-term durability of an extra-anatomical bypass in younger patients remains a consideration, our case demonstrates excellent midterm results at the 5-year follow-up. Lifelong surveillance is warranted to monitor graft patency and detect potential late complications.

## Conclusions

Delayed post-traumatic SAPs are rare but potentially life-threatening entities that require individualized management. In anatomically complex cases with insufficient landing zones, hybrid repair combining supra-aortic debranching and endovascular exclusion offers a durable and safe solution. This case demonstrates excellent long-term outcomes at the 5-year follow-up, supporting the role of hybrid techniques in selected patients.

## Data availability statement

All data generated or analyzed during this study are included in this published article.

## Funding

None.

## Disclosures

None.
